# Synergy of cell–cell repulsion and vacuolation in a computational model of lumen formation

**DOI:** 10.1098/rsif.2013.1049

**Published:** 2014-03-06

**Authors:** Sonja E. M. Boas, Roeland M. H. Merks

**Affiliations:** 1 Life Sciences Group, Centrum Wiskunde and Informatica (CWI), Amsterdam, The Netherlands; 2 Netherlands Consortium for Systems Biology, Netherlands Institute for Systems Biology, Amsterdam, The Netherlands; 3 Mathematical Institute, Leiden University, Leiden, The Netherlands

**Keywords:** lumen formation, cellular Potts model, angiogenesis, computational modelling, vacuolation, cell–cell repulsion

## Abstract

A key step in blood vessel development (angiogenesis) is lumen formation: the hollowing of vessels for blood perfusion. Two alternative lumen formation mechanisms are suggested to function in different types of blood vessels. The *vacuolation* mechanism is suggested for lumen formation in small vessels by coalescence of intracellular vacuoles, a view that was extended to extracellular lumen formation by exocytosis of vacuoles. The *cell–cell repulsion* mechanism is suggested to initiate extracellular lumen formation in large vessels by active repulsion of adjacent cells, and active cell shape changes extend the lumen. We used an agent-based computer model, based on the cellular Potts model, to compare and study both mechanisms separately and combined. An extensive sensitivity analysis shows that each of the mechanisms on its own can produce lumens in a narrow region of parameter space. However, combining both mechanisms makes lumen formation much more robust to the values of the parameters, suggesting that the mechanisms may work synergistically and operate in parallel, rather than in different vessel types.

## 1. Introduction

Blood vessels are essential for efficient transport of oxygen and nutrients throughout the body. During embryogenesis, a system of blood vessels is formed. Remodelling of the vasculature continues throughout our entire life. Sprouts of endothelial cells grow out from the existing vasculature to reach oxygen-deprived regions, including wounds or tumours, in a process called angiogenesis [1]. Subsequently, the new sprout hollows to allow blood to perfuse [2,3]. This hollowing is called lumen formation, and can occur in the absence of blood pressure, such as *in vitro* [3–6] and also *in vivo* in intersegmental vessels (ISVs) of zebrafish [6]. Which mechanisms are responsible for lumen formation is debated [7–12].

**Figure 1.**
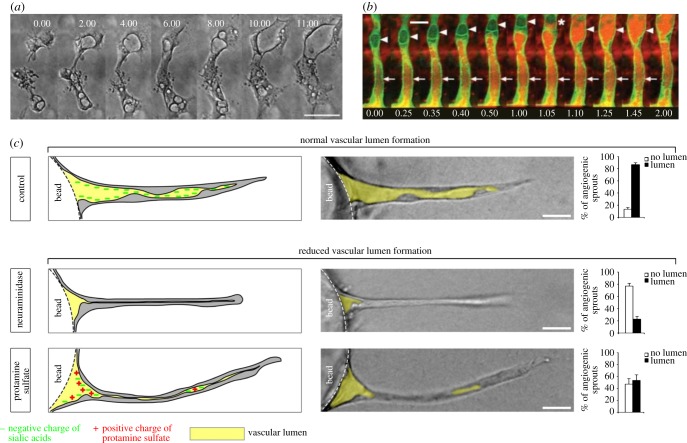
Experimental observations of lumen formation. (*a*) Time-lapse images of endothelial cells (ECs) in a three-dimensional collagen gel, showing formation of vacuoles into larger intracellular compartments. (Adapted from Kamei *et al.* [3].) (*b*) Two-photon time-lapse imaging of an ISV in which membranes are labelled with EGFP-cdc42wt and intravascularly injected red quantum dots are serially transferred by vacuole fusion. (Adapted from Kamei *et al.* [3].) (*c*) Lumens form during *in vitro* three-dimensional angiogenic sprouting assays by cell–cell repulsion facilitated by negatively charged CD34-sialomucins (control panel). Cleavage (neuraminidase panel) or neutralization (protamine sulfate panel) hereof reduces lumen formation. (Adapted from Strilic *et al.* [5].)

Lumen formation is extensively studied in epithelial tissues, which has resulted in a range of potential mechanisms [13]. Three of these can form lumens in cords of cells: cavitation, cell hollowing and cord hollowing. Cavitation is unlikely to play a role in lumen formation of endothelial tubes, where apoptosis is rarely observed [3]. The two remaining mechanisms assume intracellular lumen formation within cells in unicellular tubes (cell hollowing) versus extracellular lumen formation between cells in multicellular tubes (cord hollowing). The debate whether lumens form intracellularly [14] or extracellularly [15] in blood vessels originates from the nineteenth century [16]. This led to two opposing views on the molecular mechanisms of lumen formation in endothelium: the vacuolation mechanism [3,4] and the cell–cell repulsion mechanism [5,17]. *The vacuolation mechanism* (figure 1*a,b*) originally suggested that an intracellular lumen is formed by coalescence of vacuoles [3,18], and this view was extended with extracellular lumen formation by exocytosis of vacuoles [6,19]. *The cell–cell repulsion mechanism* (figure 1*c*) assumes that extracellular lumens initiate by active repulsion of adjacent cells and are expanded by active cell shape changes [5,17]. As vacuoles are often observed in ISVs, but not in aortae, the vacuolation mechanism is suggested to function in small vessels and cell–cell repulsion is suggested to function in large vessels [6,9–12].

It is difficult to distinguish the two proposed lumen formation mechanisms experimentally, because they use similar proteins and pathways—everything is intertwined. Therefore, we used a computational model, which allows us to isolate, modify and study single mechanisms and selected components [20–22], and compared the efficiency of both lumen formation mechanisms separately and combined. An extensive parameter sensitivity analysis of the model suggests that lumen formation is most robust to inhibitions of either mechanism when the two mechanisms are combined, indicating that they function synergistically in lumen formation. Thus, our model challenges the view that each of the mechanisms operates on its own in different types of blood vessels [6,9–12], and supports the idea that the different mechanisms operate in parallel [7].

## 2. Results

**Figure 2.**
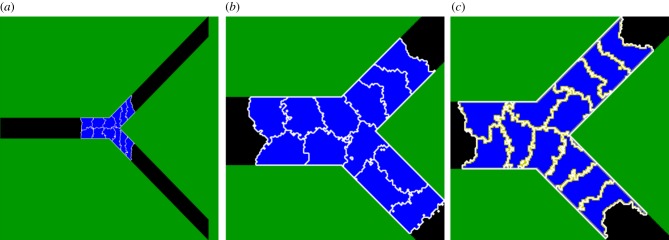
Model initialization. (*a*) The configuration of the model at initialization. (*b*) Final outcome of a simulation without cell surface polarization. (*c*) Final outcome of a simulation with cell surface polarization.

We developed a two-dimensional, multi-scale, agent-based computational model in which lumen formation emerges from predefined behaviour of components at the cellular and subcellular scale. During angiogenesis, lumens form shortly after new sprouts have originated [2,3]. Thus we can assume that sprouting and lumen formation are separated in time, such that we can model lumen formation in a preformed sprout. The sprout is represented as a branched cord of 12 cells within an extracellular matrix (ECM; figure 2*a,b*). The bifurcating geometry contains narrow, single-cell-wide regions at the tips of the branch and two-cell-wide regions at the trunk of the branch. The model currently neglects degradation or secretion of ECM by the cells that we considered in previous work [23]. ECM fluid is present at each end of the branch to allow stretching rather than widening (as seen in experiments; figure 1*a,c*) of the vessel during lumen formation.

The model represents a two-dimensional cross section of the vessel. In a cross section, a cell with an intracellular lumen will be split up, whereas it would be doughnut-shaped in three dimensions. Despite this obvious disadvantage of using a two-dimensional simulation, it still gives us useful insights into the local mechanisms driving lumen formation. Furthermore, the computational efficiency of the two-dimensional model allows us to evaluate the behaviour of the model for a large number of parameter settings.

**Figure 3.**
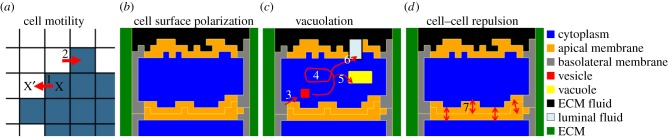
Modules of the computational model. (*a*) Cell motility by a copy of the membrane outwards (1) and inwards (2). (*b*) Cell surface polarization, resulting in cytoplasm (blue) surrounded by a basolateral membrane (grey) and an apical membrane (orange). (*c*) Vesicles (red) can be created by pinocytosis (3), move in the cell by a biased random walk (4), fuse into vacuoles (yellow) (5) and be secreted (6) into luminal fluid (light blue). (*d*) The apical membranes of different cells repulse each other (7) owing to the negative charges on the extracellular domains of the CD34-sialomucins herein.

The model consists of four modules (figure 3) that we combine differently to represent each of the mechanisms. *Cell motility* is regulated by the cellular Potts model (CPM) [24,25], which considers the shape of cells and their adhesive properties. Agent-based extensions of the CPM represent subcellular structures, including membrane proteins, vesicles and vacuoles. *Cell surface polarization* results in a basolateral membrane that lines the vessel and connects the cells, and an apical membrane where the lumen will form. During *vacuolation*, pinocytotic vesicles are formed within the cells, coalesce into vacuoles and are secreted at the apical membrane. *Cell–cell repulsion* occurs by active repulsion of apical membranes from opposing cells. We briefly describe each module here; for details and reference settings (tables 1–3) see Material and methods.

**Table 1.** Reference values of external contact energy. The external contact energy (*J*_E_) is listed for each type combination.

	cytoplasm	basolateral	apical	vesicle	vacuole	ECM	ECM fluid	luminal fluid
cytoplasm	10							
basolateral	10	30						
apical	200	50	200					
vesicle	10	10	10	10				
vacuole	10	10	10	10	10			
ECM	130	10	10	10	10	10		
ECM fluid	10	200	50	10	10	130	0	
luminal fluid	10	200	50	10	10	130	0	0

**Table 2.** Reference values of internal contact energy. The internal contact energy (*J*_I_) is listed for each type combination.

	cytoplasm	basolateral	apical	vesicle	vacuole	ECM	ECM fluid	luminal fluid
cytoplasm	10	5	5	10	20	—	—	—
basolateral		10	70	100	100	—	—	—
apical			10	1	1	—	—	—
vesicle				10	5	—	—	—
vacuole					5	—	—	—
ECM						—	—	—
ECM fluid							—	—
luminal fluid								—

**Table 3.** Reference parameter values. The reference value is listed for parameters that relate to probabilities, the motility *μ* and the elasticity *λ* for certain types.

*P*_A_ = 1.0	*P*_fuse_ = 1.0	*P*_pin_ = 1.0	*μ* = 50	*λ*_cell_ = 7	*λ*_fluids_ = 6	*λ*_vacuole_ = 50	*λ*_vesicle_ = 1000

### 2.1. Cell motility module

The CPM [24,25] is a convenient model to deal with structural and spatial aspects in lumen formation, because it considers the shape of cells and their physical interactions with their surroundings. Cells are represented as patches of connected sites of a lattice. The diameter of a cell is approximately 5–10 μm (one pixel = 250 nm), and a cell is initialized with 625 pixels (±25 × 25). We represent the subcellular scale using a compartmentalized CPM [26–28]. Each lattice site 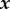 in a compartment is associated with the same unique compartment identifier (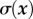). Depending on the mechanism that is tested, compartments with the type (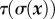) *cytoplasm*, *apical membrane*, *basolateral membrane*, *vesicle* and *vacuole* can exist within a cell. ECM, ECM fluid and cells have an additional unique cell identifier 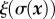, with the set of compartments *σ* that belong to the same cell having the same cell identifier. The ECM is immobile, but cells and compartments move by copying pixels at the membrane inwards or outwards (figure 3*a*). The source pixel (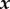) together with a random, adjacent target pixel (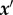) is randomly selected for each copy attempt, resulting in a stochastic simulation. Acceptance of these pixel copies depends on properties of the cells and compartments, such as adhesion/repulsion strength (contact) and size (area). Adhesion or repulsion strength can result from protein interactions and from surface tensions on membranes and is modelled by contact energy. A distinction is made between internal contact energy between compartments of the same cell (*J*_I_(*τ*,*τ*)) and external contact energy between compartments of different cells (*J*_E_(*τ*,*τ*)). As a reference contact energy, we use a value of *J* = 10: a lower contact energy leads to adhesion, whereas a higher contact energy leads to repulsion. Lattice sites that are not occupied by ECM or cells are of type *ECM fluid* or *luminal fluid*. ECM fluid is already present at initialization next to the branch and is in contact with the ECM. Luminal fluid is secreted by vesicles and vacuoles in between cells. Luminal fluid will fuse to the ECM fluid when surrounded by ECM fluid or when in contact with the ECM. Cells, vesicles, vacuoles and subvolumes of fluid have a preferred size (*A*), which is conserved on average, with the elasticity parameter *λ*_type_ (see Material and methods for details) regulating the allowed deviation from the preferred size [24]. For cells, vesicles and vacuoles, this reflects the semipermeability of membranes for water. For subvolumes of fluids, this results in near incompressibility, which could resemble a hydrostatic pressure in the sublumens.

### 2.2. Cell surface polarization module

Cell surface polarization into a basolateral and an apical domain is the first step in lumen formation and is regulated by integrin signalling from the ECM [9,17,29–33]. In the computational model, the membrane (re)polarizes every other time step into an apical and a basolateral compartment based on the relative position to the ECM (figures 2*c* and 3*b*). During repolarization, each ‘mispolarized’ pixel in the membrane is assigned to the correct compartment. A membrane pixel becomes part of the basolateral compartment when it is in direct contact with the ECM. To form the lateral junctional regions of the cells, membrane pixels that have at least two neighbouring pixels that are in direct contact with the ECM also become part of the basolateral compartment. The rest of the membrane pixels become part of the apical compartment. Contact energy is used to mimic adherens junctions (*J*_I_ (*apical*, *basolateral*)) and to set the surface tension of the apical membrane (*J*_E_ (*fluids*, *apical*)).

### 2.3. Vacuolation module

The vacuolation mechanism is one of the two proposed mechanisms for lumen formation in blood vessels [3,18]. Vacuoles are often observed *in vitro* and *in vivo* during lumen formation as summarized by Davis & Bayless [4]. Vacuoles were observed with electron microscopy (figure 1*a*) and by expression of the green fluorescent protein (GFP) fusion proteins GFP-Rac1, GFP-Cdc42 and Moesin1-EGFP, which colocalize with the vacuoles [4,34–36]. Vacuoles show highly dynamic behaviour, as they continuously fuse together, grow, shrink and disappear [3,6]. Kamei *et al.* [3] showed that a label (carboxyrhodamine) added to the medium is taken up into the vesicles by pinocytosis and is transferred to vacuoles by fusion of vesicles into vacuoles. Fusion might be facilitated by caveolin-1 because it concentrates at vacuole–vacuole contact areas [4]. Pinocytotic vesicles are probably trafficked along microtubules and actin filaments [37]. Targeting to the apical membrane might involve Cdc42 and Moesin1 [4,6], which both co-localize with vesicles and have a high affinity for phospholipids specific for the apical membrane. Eventually, vesicles and vacuoles bridge the entire cell [3] and are exported at the apical membrane through exocytosis to create a fluid-filled space for a lumen [19]. Each of these steps is explicitly modelled in this module.

To mimic pinocytosis, we assumed that if a membrane pixel becomes internalized in the cell owing to cell movements, it has a probability (*P*_pin_) to become a new compartment of type vesicle (figure 3*c*; step 3) or to become part of the cytosol. The vesicle diameter is approximated at 250 nm [38], which is equal to one pixel length in the model. This size is achieved by means of a target area of 1 and a high inelasticity (*λ*_vesicle_). Vesicle transport is precisely regulated with vesicle-associated motor proteins that walk along microtubules and actin filaments [37], which gives rise to stochastic motion of vesicles if the cytoskeleton is randomly oriented [37–39]. Random binding and unbinding of vesicles and associated motor proteins with a randomly oriented cytoskeleton leads to diffusive transport behaviour [39]. In our model, we therefore model vesicle transport by a random walk with stepping probability *P*_A_ (see Material and methods), biased by preferential adhesion to the apical membrane and fusion into vacuoles (figure 3*c*; step 4). Vesicles preferentially adhere to the apical membranes, vesicles and vacuoles by considering their contact energy in their stepping. Fusion of neighbouring vesicles, vacuoles or a combination of the two happens with probability *P*_fuse_ (figure 3*c*, step 5) and generates a single vacuole compartment for which the target areas are combined. A vacuole moves by means of the usual CPM rules (see Material and methods) and is only restricted by its contact energy and size constraint. When a single pixel of type vacuole is no longer in contact with vesicles or vacuoles, it becomes a vesicle. Secretion occurs when a vesicle or vacuole pixel is in the apical membrane (figure 3*c*; step 6). This pixel, together with all connected pixels of type vesicle or vacuole, is secreted to form a fluid-filled luminal space of type *luminal fluid*. The cell membrane is subsequently repolarized, which also leads to an increase in the size of the apical membrane.

### 2.4. Cell–cell repulsion module

The alternative cell–cell repulsion mechanism [5,17] assumes active, electrostatic repulsion of the apical membranes of adjacent cells, followed by active cell shape changes to extend the lumen. Previously, it was suggested that lumens can form by relocalization of junctional complexes over the cell membrane, leading to local differences in cell adhesion at the future luminal and basolateral sides of cells [40–42]. In addition to such differential adhesion-driven lumen formation, Strilic *et al*. [5,17] showed in the developing mouse aorta and in a three-dimensional angiogenic sprouting assay that apical membranes actively repulse each other during lumen formation by expression of CD34-sialomucin glycoproteins, such as PODXL. These transmembrane glycoproteins have negatively charged extracellular domains and are transported to the apical membrane in preformed vesicles.

This active repulsion is modelled with high contact energy between apical membranes of adjacent cells (*J*_E_ (*apical*, *apical*); figure 3*d*; step 7). Pixels of type cytoplasm can be at the membrane, because cell surface polarization is only performed every other time step in the model to allow some freedom of movement for the membranes and for internalization of membrane pixels for pinocytosis. Therefore, *J*_E_ (*cytoplasm*, *apical*) is set to the same value. *J*_E_ (*apical*, *apical*) and *J*_E_ (*cytoplasm*, *apical*) together will now be referred to as *J*_rep_. Note that this module of cell–cell repulsion only includes short-range, electrostatic membrane repulsion owing to CD34-sialomucin glycoproteins. The additional mechanisms in our model, including cell shape changes and invasion of luminal fluid, may act to further separate the membranes from each other.

### 2.5. Reconstruction of the mechanisms

Each lumen formation mechanism can be reconstructed from the four modules: cell motility, cell surface polarization, vacuolation and cell–cell repulsion. The first three modules are combined to study the vacuolation mechanism, the first two and last for the cell–cell repulsion mechanism and all four modules together to study them combined, ‘the combined mechanism’. A simulation is run for 10 000 time steps to allow the creation of continuous lumens. During one time step, the Monte Carlo step (MCS), as many copy attempts as there are pixels in the grid are attempted. Continuous lumens are formed in approximately 24 h [4,5]; a time step in the model thus corresponds to a few seconds in real time.

**Figure 4.**
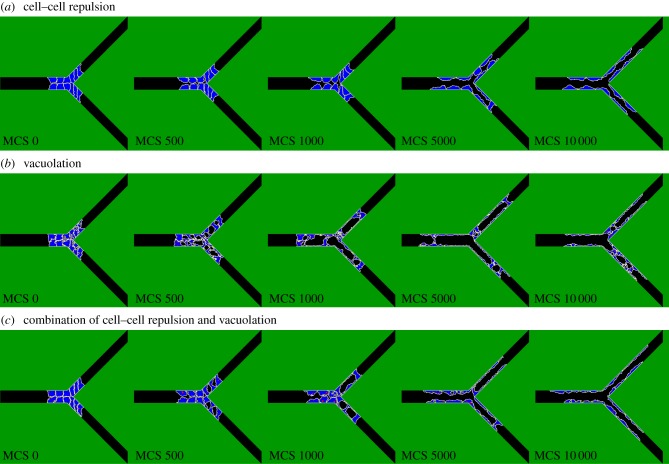
Time-lapse images of lumen formation by each mechanism. (*a–c*) A time-lapse image of lumen formation by cell–cell repulsion, vacuolation and the combined mechanism for reference setting, respectively.

Because the model has a strong stochastic component, we run each simulation 30 times. Efficiency of lumen formation is expressed as the fraction of simulations that have formed a continuous lumen at the end of a simulation (continuity fraction). The lumen is continuous when the fluid in the three tips of the branch is part of one single connected component. Figure 4 shows a time-lapse image for each mechanism at reference settings (corresponding to the electronic supplementary material, videos S1–S3). During cell–cell repulsion (e.g. figure 4*a*, MCS 10 000), the cells stretch and flatten along the ECM, very similar to the experimental pictures in figure 1*c*. The shape and size of the sublumens that are formed during vacuolation (e.g. figure 4*b*, MCS 500) visually resemble the experimental observations of Kamei *et al.* [3] (figure 1*a*). Although lumens do not form by vacuolation at reference settings, they do form for higher pinocytosis rates. The model is robust to practically all changes in parameters that describe the transport and fusion of vesicles and vacuoles (see electronic supplementary material, figure S1 and text S1).

Ideally, all parameters in the model would have a quantitative value derived from experiments to allow comparison. However, most values are not known experimentally. Notably, Klann *et al*. [43] point out that they could not quantify time constants of reactions and transport processes in vesicle dynamics, because present experimental results focus on the functional and qualitative identification of molecular interactions and pathways rather than on the dynamics of the system. Fortunately, our computational model can still be validated in a qualitative manner. Most parameters in the lumen formation model correspond to a protein or molecular process in lumen formation. As a result, qualitatively reducing the value of a parameter corresponds to a molecular knockout or inhibition of the molecular mechanism it represents and should produce similar effects.

### 2.6. Vacuolation requires a high pinocytosis rate

In experiments, inhibition of pinocytosis prevents lumen formation and vacuole formation. Pinocytosis is an integrin-dependent process and is inhibited during these experiments by blockage of these sites (α2β1 in collagen matrices [18] or αvβ3 and α5β1 in fibrin–fibronectin matrices [44]).

**Figure 5.**
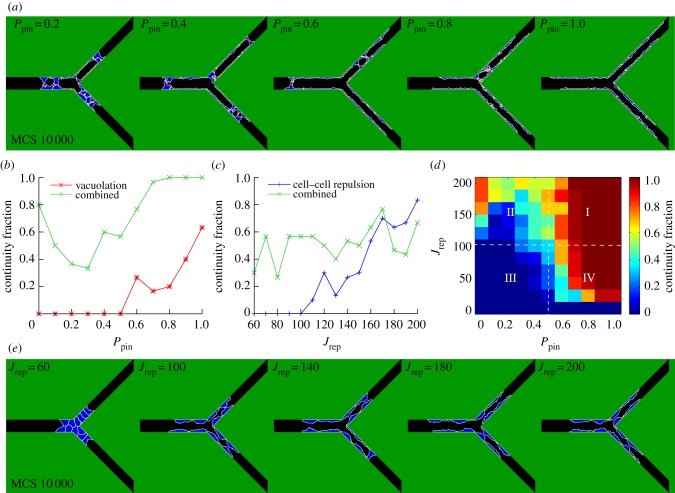
Robustness and synergy. (*a*) Lumen formation by vacuolation at MCS 10 000 for different values of the pinocytosis rate (*P*_pin_). (*b*) Continuity fraction of the combined mechanism and the vacuolation mechanism as a function of *P*_pin_. (*c*) Continuity fraction of the combined mechanism and the cell–cell repulsion mechanism as a function of the repulsion strength (*J*_rep_). (*d*) The continuity fraction of the combined mechanism expressed in a heat map as a function of *P*_pin_ and *J*_rep_. (*e*) Lumen formation by cell–cell repulsion at MCS 10 000 for different values of *J*_rep_.

To mimic this blockage, we reduced the rate of pinocytosis (*P*_pin_) in our model. In agreement with the experiments, continuous lumens formed by vacuolation for high pinocytosis rates, but not for low rates (figure 5*a,b*).

### 2.7. Cell–cell repulsion requires a high cell–cell repulsion strength

Reduction of the cell–cell repulsion strength by cleavage or neutralization of the negative extracellular charged domains of CD34-sialomucins also inhibits lumen formation [5]. Lumen formation is rescued by subsequent addition of negatively charged dextran sulfate that binds to cell surfaces.

To mimic the cleavage/neutralization in our model, we reduced the repulsion strength (*J*_rep_). As seen in the experiment, continuous lumens formed by cell–cell repulsion for high repulsion strengths, but not for low values of this parameter (figure 5*c,e*).

### 2.8. Vacuolation and cell–cell repulsion combined produce lumens most robustly

Figure 5*b,c* shows that lumen formation by the combined mechanism (green curve) is more robust to changes in the pinocytosis rate than the vacuolation mechanism (red curve) and that the combined mechanism is more robust to changes in the repulsion strength than the cell–cell repulsion mechanism (blue curve), respectively. The combined mechanism already has a continuity fraction of 0.8 in the absence of pinocytosis (figure 5*b*). The continuity fraction of the combined mechanism is 0.3 at *J*_rep_ = 60 compared with a continuity fraction of zero for the cell–cell repulsion mechanism (figure 5*c*, *p*-value = 1.1 × 10^−3^).

The robustness of the combined mechanism to inhibition of the pinocytosis rate and to inhibition of the cell–cell repulsion strength disagrees with the experiments discussed above, in which inhibition of either mechanism reduced lumen formation. This discrepancy between our model and the experimental observations could be caused by the fact that the mechanisms of vacuolation and cell–cell repulsion are likely to be intertwined, while they are completely separated in the model. First, blockage of integrins to inhibit pinocytosis could also affect cell surface polarization, which is a crucial step for cell–cell repulsion. Second, cleavage or neutralization of the negative extracellular charged domains of CD34-sialomucins can affect the cytoskeleton, and thereby might also affect the transport and exocytosis of vesicles and vacuoles at the apical membrane. The intracellular domains of CD34-sialomucins are linked to the cytoskeleton, which disengages upon neutralization of the extracellular domains [45]. Thus, the discrepancy between the combined mechanism and experiments does not necessarily mean that the combined mechanism is incorrect, but could reflect the high level of cross talk between the mechanisms that drive vacuolation and cell–cell repulsion.

### 2.9. Synergy of vacuolation and cell–cell repulsion

We next asked whether the robustness of the combined mechanism is due to synergy of vacuolation and cell–cell repulsion. Figure 5*d* shows the combined effect of the pinocytosis rate *P*_pin_ and the repulsion strength *J*_rep_ on the continuity fraction of the combined mechanism. Lumens are practically never continuous for low values of *P*_pin_ and *J*_rep_ (quadrant III), and practically always continuous for high values of both (quadrant I). The high continuity fractions in quadrant IV indicate that vacuolation (dependent on *P*_pin_) reinforces cell–cell repulsion (dependent on *J*_rep_) in lumen formation. Similarly, the high continuity fractions in quadrant II indicate that cell–cell repulsion reinforces vacuolation in lumen formation.

How does this synergy arise in the model? The cell–cell repulsion mechanism assists the vacuolation mechanism by breaking barriers, formed by adhering cells, between the sublumens. Additionally, cell–cell repulsion enlarges and stabilizes small sublumens that are created by secretion of vacuoles between cells. The vacuolation mechanism also reinforces the cell–cell repulsion mechanism. In the absence of vacuolation and for low cell–cell repulsion strength, cells do not detach, and the vessel thus remains solid. An increase in the pinocytosis rate (quadrant IV) results in the formation of sublumens, which drive cells to reposition into a multicellular, overlapping configuration. Cells that become overlapping can now detach by cell–cell repulsion in combination with secretion of vacuoles between the overlapping cells. Single cells that span the vessel can become pierced by vacuolation to create a continuous lumen. However, cell–cell repulsion for high repulsion strengths (figure 5*d*, *J*_rep_ > 140) is more efficient in the absence of pinocytosis than for low pinocytosis rates. For the latter, cells do not get the chance to overlap, as will be explained in §2.10.

### 2.10. Cells in unicellular sprouts need to reposition for cell–cell repulsion

*A priori*, we had expected that cell–cell repulsion could only generate a continuous lumen in multicellular tubes and not in tubes with linearly, head-to-tail arranged cells such as in the tips of the branch. However, the cells in the branch tips repositioned to an overlapping (brick-like), multicellular configuration, in line with experimental observations [19,46], and this overlap allows lumen formation by cell–cell repulsion. We found that this repositioning is driven by two counteracting forces between cells: strong attachment and active cell–cell repulsion. Cells do not immediately detach from each other upon active cell–cell repulsion as the standard CPM model treats the attachment of adjacent cell membranes as a vacuum. The membranes must zipper apart to let the fluid seep in. In the presence of vacuolation (figure 5*d*, *J*_rep_ > 140), fluid is created between cells, and cells use cell–cell repulsion to instantly detach rather than to overlap.

**Figure 6.**
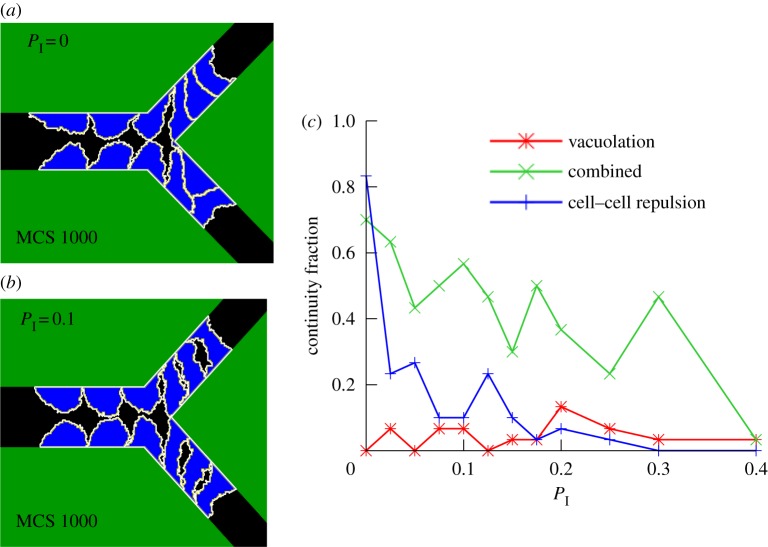
Effect of cell overlap. (*a*) Enlarged view of overlapping cells for *P*_I_ = 0. (*b*) Enlarged view of reduced overlapping capacity for *P*_I_ = 0.1. (*c*) Continuity fraction of all three mechanisms as a function of *P*_I_.

To check whether this zippering affects the efficiency of lumen formation by the cell–cell repulsion mechanism, we adapted the CPM to allow de novo insertion of ECM fluid [47]. With probability *P*_I_, we consider the change in effective energy (Δ*E*) resulting from ECM fluid insertion 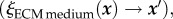 rather than from the extension of the cell membrane 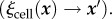 In this extended CPM, the original CPM is recovered for *P*_I_ = 0. Higher values of *P*_I_ allow cells to insert ECM fluid in between the repulsive apical membranes. Figure 6*a,b* shows magnifications of the simulations at MCS 1000 for *P*_I_ = 0 (figure 4*a* and the electronic supplementary material, video S1) and for *P*_I_ = 0.1 (see electronic supplementary material, video S4), respectively. These magnifications show that cells overlap in the tips of the branch for *P*_I_ = 0 and not for *P*_I_ = 0.1. Fluid insertion between cells thus seems to reduce overlap of cells, and thereby prevents lumen formation in the tips of the branch (see the electronic supplementary material, figure S2, for quantified results).

We next studied the robustness of each mechanism to changes in the value of *P*_I_. Figure 6*c* shows that the combined mechanism (green curve) is more robust to changes in *P*_I_ than the cell–cell repulsion mechanism (blue curve). The vacuolation mechanism (red curve) was not continuous for reference settings and this does not significantly change for higher values of *P*_I_. Notably, the continuity fraction of the combined mechanism is often higher than the continuity fraction of the cell–cell repulsion mechanism and the vacuolation mechanism combined (e.g. for *P*_I_ = 0.3, *p*-value = 1.1 × 10^−4^), another indication of reinforcement.

### 2.11. Vacuolation requires impermeable vessel walls

The vacuolation mechanism and the cell–cell repulsion mechanism assume a different permeability of cells for fluid. Kamei *et al*. [3] showed that red quantum dots in lumens, formed by vacuolation, do not mix with the extracellular environment. The red quantum dots serially transferred from the dorsal aorta to previously unlabelled vacuolar compartments of ISVs in zebrafish. By contrast, the cell–cell repulsion mechanism assumes open connections through paracellular openings and fluid can flow into the lumen from the ECM [17]. To mimic the effect of permeable vessel walls, we tested the behaviour of the model for *λ*_fluids_ = 0. In this case, the fluids become completely ‘compressible’. Only the vacuolation mechanism requires fluid incompressibility for generating continuous lumens (see electronic supplementary material, figure S3), because the lumens otherwise continuously collapse. Thus, our simulations suggest that vessel walls must be impermeable to water (or lumen fluid must be actively replenished to maintain hydrostatic pressure) in order to generate continuous lumens by the vacuolation mechanism, but this is not required for the cell–cell repulsion mechanism or for the combined mechanism.

## 3. Discussion

Extensive experimental research has resulted in two alternative proposed mechanisms of lumen formation: vacuolation [3,4] and cell–cell repulsion [5,17]. Our computational model suggests that the two mechanisms may act synergistically in lumen formation. Cell–cell repulsion can reinforce vacuolation by stabilizing sublumens and by separating cells to connect sublumens with the ECM fluid. Vacuolation can reinforce cell–cell repulsion by creating sublumens, which repositions cells into an overlapping configuration, and by piercing single cells in the tips of the branch. Additionally, vacuolation could assist in the expansion of the lumen, which was so far suggested in the cell–cell repulsion mechanism to occur by cell shape changes.

We validated our model assumptions and simulation results based on published experimental evidence. As previously discussed, for lack of quantitative values of the model parameters, the model can only make qualitative predictions. First, without apical–basolateral cell surface polarization, no lumens are formed in our model (figure 2*b*). This agrees with experiments in which polarization was prohibited in the absence of functional vascular endothelial (VE)-cadherin or phosphatase and tensin homologue [6,17]. Second, in our simulations of the vacuolation mechanism, lumens cannot form for low pinocytosis rates (figure 5*b*). Experimentally, lumen formation is indeed prevented by blockage of integrin signalling for pinocytosis [18,44]. Third, in our simulations of the cell–cell repulsion mechanism, continuous lumens cannot form at low repulsion strengths (figure 5*c*). In agreement with this model prediction, neutralization or cleavage of the extracellular negative domains of CD34-sialomucins reduces lumen formation [5].

**Figure 7.**
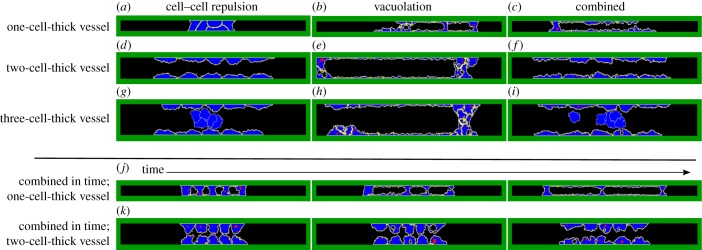
Lumen formation by each mechanism in different vessel types. (*a–i*) Lumen formation by cell–cell repulsion, vacuolation or the combined mechanism from left to right for an initial vessel thickness of one cell (top row), two cells (second row) or three cells (third row) at MCS 10 000. (*j,k*). Time-lapse images (MCS 500, 1000 and 2000) of lumen formation by the combined mechanism in a one-cell-thick vessel and in a two-cell-thick vessel.

Although the model simulations suggest that the vacuolation and repulsion mechanisms act synergistically, one may ask whether both mechanisms indeed co-occur *in vivo*. It is possible that vacuolation and cell–cell repulsion function in different types of vessels; vacuolation for single-cell capillaries (e.g. ISV of zebrafish) and cell–cell repulsion for multicellular tubes (e.g. dorsal aortae of mice) [3,6,9–12]. Therefore, we tested how the vacuolation, cell–cell repulsion and combined mechanisms would act in different types of vessels, which we represent in our model by initial configurations of one, two and three layers of cells (figure 7). Cell–cell repulsion is not functional in a one-cell-thick vessel with aligned cells (figure 7*a*). Cells do start to overlap, but not sufficiently. Vacuolation does create a lumen, but it continuously collapses again (figure 7*b*). The combined mechanism forms stable lumens (figure 7*c*). In two-cell-thick vessels cell–cell repulsion is much more efficient than vacuolation (figure 7*d–f*) and stable lumens form by cell–cell repulsion and by the combined mechanism, but not by vacuolation. In three-cell-thick vessels, the cell–cell repulsion and combined mechanisms can reproduce cavitation, the apoptosis of cells in the middle of the vessel that detached from the vessel wall (figure 7*g,i*), which is often seen in epithelium [13]. Interestingly, the combined mechanism produces phenomena that optically resemble aspects of vacuolation or of cell–cell repulsion, depending on whether it acts in one-cell-thick vessels or in two-cell-thick vessels. The optical resemblance to vacuolation is illustrated in figure 7*j* and the resemblance to cell–cell repulsion in figure 7*k*, which show the temporal development of lumen formation by the combined mechanisms in a one-cell-thick vessel and a two-cell-thick vessel, respectively. Thus, although vacuolation is observed in capillaries and cell–cell repulsion in multicellular tubes, the combined mechanism could be the underlying mechanism in both vessel types.

To validate whether the combined mechanism is actually functional *in vivo*, we propose the following experiments. Based on our modelling results in figure 7, we expect that inhibition of vacuolation by reducing pinocytosis should prevent lumen formation in capillaries (as in figure 7*a*), but not in larger vessels (as in figure 7*d,g*), and inhibition of cell–cell repulsion by cleavage of negatively charged extracellular proteins should destabilize lumen formation in all vessel types (as in figure 7*b*,*e*,*h*). Here, simply visualizing vacuoles microscopically will not suffice for validating the combined mechanism: lumens also form with cell–cell repulsion in combination with secretion of pinocytotic vesicles, without fusion of vesicles into vacuoles, depending on the amount of vesicles (see electronic supplementary material, figure S4).

Besides synergy of the vacuolation and cell–cell repulsion mechanisms, our model also predicts that lumen formation by cell–cell repulsion in unicellular tubes requires cells to reposition into a (brick-like) overlapping, multicellular configuration. Our model results suggest that cell–cell adhesion along with strong cell–cell repulsion strengths facilitate the formation of such overlapping cellular configurations. To validate this mechanism, it should be established experimentally if cells immediately detach once CD34-sialomucins are in the apical membrane, or that strong adhesion keeps them attached for time spans sufficiently long to allow repositioning of cells.

These validation experiments all result from qualitative model predictions, as the quantitative values for most parameters are not known. Dose-dependent experiments for cell adhesion strengths, pinocytosis rates and cell–cell repulsion strengths can help to tune the representing qualitative parameters in the model. Additionally, various parameters could be quantified experimentally to allow for quantitative model predictions. The adhesion strength of cells can be quantified by the force that is required to pull them apart [48]. This method might also be useful to find quantitative values, or at least the relative ordering, of the contact energy parameters that describe adhesions between polarized cells, non-polarized cells and possibly also the ECM. Other contact energy parameters of our model describe adhesions of subcellular compartments such as vesicles, which cannot be quantified in this way. Instead, vesicles and vacuoles can be visualized microscopically and their speed and type of movement (e.g. diffusive) could thus be quantified.

Our model can become a useful tool for designing new experiments and new insights into lumen formation. We propose three new research questions in which we believe cooperative computational and experimental research is important. First, what is the exact function of several key proteins in lumen formation? Cdc42 and Moesin1 are, for instance, suggested to be involved in polarization of the cell, in targeting of vesicles to the apical membrane and in structural changes of the cytoskeleton for cell shape changes [6,17,29]. It is difficult to pinpoint their exact function by experiments only, because lumen formation fails all together in the absence of these proteins. Second, how is lumen formation regulated in dynamically growing sprouts? To focus on lumen formation, we started our model with a preformed sprout. To gain insights into the regulation of angiogenesis as a whole, the model could be extended with ECM remodelling and dynamic sprouting. Third, if the combined mechanism, indeed, drives lumen formation, then how are the two mechanisms regulated and balanced to locally optimize lumen formation? For each question, the model can be used to test consistency of hypotheses, which can provide new insights and help to guide new experiments. In conclusion, in collaboration with experimentalists, our simulation model can contribute to a better understanding of the mechanisms of lumen formation during blood vessel development.

## 4. Material and methods

We developed an agent-based, computational model of lumen formation that connects the subcellular, cellular and the ‘vessel’ scales. The CPM [24,25] describes the motility, shape and physical interactions of cells. To model polarization of the cell surface and the creation of fluid-filled vesicles and vacuoles, we use an extension of the CPM in which cells can compartmentalize [26–28]. In this section, we first explain the extended version of the CPM. Next, we outline the modelling of pinocytosis, vesicle and vacuole movement and secretion.

### 4.1. Compartmental cellular Potts model

**Figure 8.**
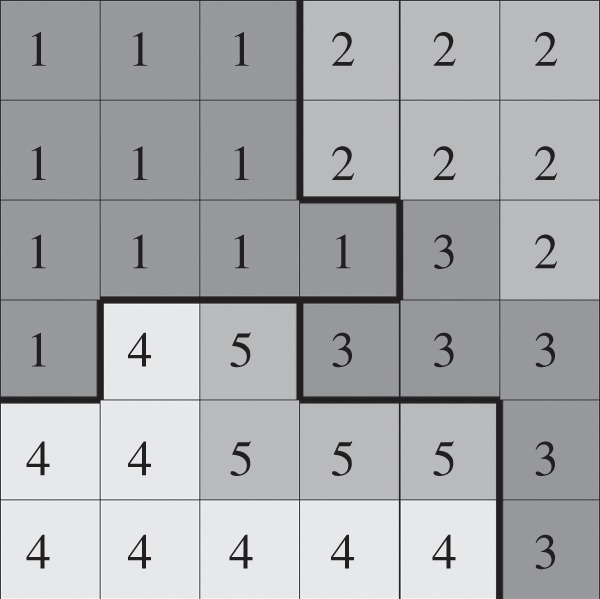
Representation of cells in the compartmental CPM. The compartmental CPM projects cells on a regular lattice and cells can consist of multiple compartments. All lattice site, 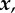 of the same cell are outlined by a bold black line, representing that they share the same unique cell identifier 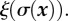 Each lattice site has a number that represents its compartment identifier 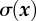 and its type 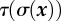 is indicated by its colour.

The CPM projects cells on a regular lattice (figure 8). Each lattice site, 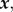 is associated with a unique compartment identifier 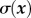 and has a type 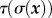, which can be *cytoplasm*, *apical*, *basolateral*, *vesicle*, *vacuole*, *ECM*, *ECM fluid* or *luminal fluid* (figure 3). Initially cells, ECM and ECM fluid consist of a single compartment with a unique cell identifier 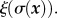 Additional compartments, which are formed in a cell upon membrane polarization and vacuolation, obtain the same cell identifier 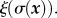 New cell identifiers with type *luminal fluid* are created upon secretion of vesicles and vacuoles.

To model random motility, fluids and subcellular compartments (except for vesicles) move by copies from 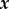 to a randomly selected second neighbouring lattice site 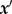 (figure 3*a*). A copy changes the effective energy of the system (*E*), which depends on the areas and contacts (adhesive properties) of cells, compartments and fluids: *E* = *E*_area_ + *E*_contact_. A copy is always accepted for a decrease in effective energy and is otherwise accepted with a Boltzmann probability 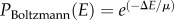, where *μ* resembles active cell motility.

*E*_area_ is the costs in energy for deviation of the actual area (*a*) from the preferred target area (*A*), with an elasticity lambda, and is given by
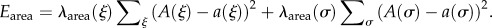
We model fluids as near-incompressible fluids; fluids are cell identifiers of type *ECM fluid* or *luminal fluid* with *λ*_area_(*ξ*) ≠ 0 and *λ*_area_(*σ*) ≠ 0.

Contact energy (*E*_contact_) mimics adhesion and repulsion at interfaces of compartments. There are two types of contact energy: internal (*J*_I_) and external (*J*_E_). Internal contact energy is defined between compartments of the same cell, and external contact energy between compartments of different cells. The total contact energy is defined as
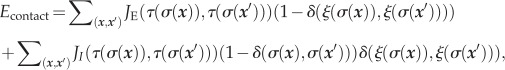
with the Kronecker delta *δ*(*x*, *y*) = {1, *x* = *y*; 0, *x* ≠ *y*}. All *J*-values are listed in tables 1 and 2. Cells (non-polarized or polarized) stay connected in the absence of lumen formation mechanisms (figure 2*b,c*) for reference *J*-values.

### 4.2. Pinocytosis

**Figure 9.**
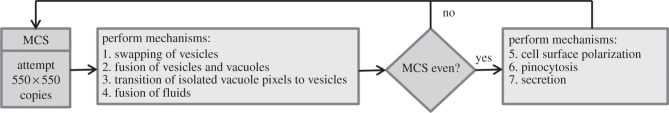
Flow chart of the model. During each time step, also called Monte Carlo step (MCS), as many copy attempts as there are pixels in the lattice (550 × 550) are attempted. After each time step, or after every other time step, additional functions for lumen formation are performed.

During each MCS, as many copy attempts as there are pixels in the lattice are performed (550 × 550). Extra mechanisms (*m*) of lumen formation are performed after every *n_m_* MCS (figure 9). Cell surfaces polarize every other MCS (figure 9; mechanism 5; *n*_5_ = 2) to allow these polarized membrane pixels to internalize by usual CPM copies, representing invagination of ECM fluid at the membrane as seen during pinocytosis. Such internalized polarized membrane pixels become compartments of type *vesicle* with probability *P*_pin_ or otherwise part of the cytoplasm (figure 9; mechanism 6; *n*_6_ = 2). A vesicle is kept one pixel in size by a target area of one and a high lambda. As ECM fluid is taken up into the vesicle during pinocytosis, the target area of ECM fluid decreases, while the target area of the pinocytosing cell increases by one.

### 4.3. Vesicle and vacuole movement

Vesicles swap position with a randomly selected neighbour once per MCS (figure 9; mechanism 1; *n*_1_ = 1). This swapping is performed with a pre-set acceptance probability *P*_A_ multiplied by *P*_Boltzmann_(*E*), with *E* the resulting effective energy of the swap. *P*_A_ tunes the velocity of the vesicle.

Fusion events of vesicles and vacuoles can occur every MCS (figure 9; mechanism 2; *n*_2_ = 1) and during a copy of a vacuole over a vesicle. Every MCS, neighbouring compartments of type *vesicle* or *vacuole* fuse with probability *P*_fuse_ into a single compartment with type *vacuole*. The target area of the formed vacuole is the sum of the target areas of the fused compartments. Similarly, when a vacuole copies over a vesicle of the same cell, the target area of the vacuole is increased by the target area of the vesicle.

As vesicles can become vacuoles by fusion, small vacuoles can also become vesicles. First, single pixels of type *vacuole* that are split off from a vacuole, called the donor vacuole, and are surrounded by cytoplasm become vesicles each MCS (figure 9; mechanism 3; *n*_3_ = 1). Second, a donor vacuole that became a single pixel in size by a copy becomes a vesicle. In both cases, the created vesicle gets a target area of one, unless the donor vacuole in question had a target area of zero. Then, the vesicle is assigned with a target area of zero and will soon be deleted by regular CPM movements. If a donor vacuole remains, then the target area of the created vesicle is subtracted from the target area of the donor vacuole. If a donor vacuole becomes one pixel in size by a copy event and has a target area larger than one, then its residual target area must be redistributed to remain a target area of one for the created vesicle. If the donor vacuole was copied over by luminal fluid or a vacuole, then the residual target area is added to that compartment. Otherwise, the residual target area is added to ECM fluid to keep the total target area of the system constant.

### 4.4. Secretion

Secretion can occur every other MCS (figure 9; mechanism 7; *n*_7_ = 2) and by a copy of a fluid over a vesicle or vacuole. Every other MCS, a pixel at the membrane of type *vesicle* or *vacuole*, together with all first-order connected pixels of type *vesicle* or *vacuole*, becomes a compartment of type *luminal fluid*. The combined target area of the simultaneously secreted vesicles and vacuoles is assigned to the luminal compartment and is subtracted from target area of the secreting cell. If only a part of the vacuole is secreted, then the size of the secreted part is subtracted from the target area of the vacuole, leaving a vacuole with a minimal target area of zero. Secretion can also occur by a copy 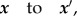 for which 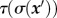 is *vesicle* and 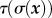 is *luminal fluid* or *ECM fluid*, resulting in a decrease of one of the target area of the secreting cell and an increase of one of the fluid. Similarly, the target area of the secreting cell decreases by one when its vesicle is copied over by a compartment of the same cell of type *apical*, *basolateral* or *cytoplasm* or by a compartment of another cell. To conserve the total target area of the system, the target area of ECM fluid is then increased by one.

ECM fluid and luminal fluids can fuse every MCS (figure 9; mechanism 4; *n*_4_ = 1) and by some copy events. Neighbouring cells of type *luminal fluid* are fused every MCS and can fuse to ECM fluid when the luminal fluid is in contact with the surrounding ECM or when it is completely surrounded by ECM fluid. When luminal fluid copies over luminal fluid of a different cell identifier and thereby deletes it, the target area of the latter is added to the first. If luminal fluid is deleted by a cell type other than *luminal fluid*, then its target area is added to ECM fluid.
